# Interferon Response in Hepatitis C Virus (HCV) Infection: Lessons from Cell Culture Systems of HCV Infection

**DOI:** 10.3390/ijms161023683

**Published:** 2015-10-07

**Authors:** Pil Soo Sung, Eui-Cheol Shin, Seung Kew Yoon

**Affiliations:** 1 Laboratory of Immunology and Infectious Diseases, Graduate School of Medical Science and Engineering, KAIST, Daejeon 34141, Korea; E-Mail: pssung49@gmail.com; 2 Department of Internal Medicine, College of Medicine, The Catholic University of Korea, Seoul 06591, Korea

**Keywords:** hepatitis C virus, interferon, interferon-stimulated genes, innate immune response

## Abstract

Hepatitis C virus (HCV) is a positive-stranded RNA virus that infects approximately 130–170 million people worldwide. In 2005, the first HCV infection system in cell culture was established using clone JFH-1, which was isolated from a Japanese patient with fulminant HCV infection. JFH-1 replicates efficiently in hepatoma cells and infectious virion particles are released into the culture supernatant. The development of cell culture-derived HCV (HCVcc) systems has allowed us to understand how hosts respond to HCV infection and how HCV evades host responses. Although the mechanisms underlying the different outcomes of HCV infection are not fully understood, innate immune responses seem to have a critical impact on the outcome of HCV infection, as demonstrated by the prognostic value of IFN-λ gene polymorphisms among patients with chronic HCV infection. Herein, we review recent research on interferon response in HCV infection, particularly studies using HCVcc infection systems.

## 1. Introduction

Hepatitis C virus (HCV) is a positive-stranded RNA virus in the family *Flaviviridae*, and it is estimated 130–170 million people are infected with HCV worldwide [1]. Acute HCV infection is spontaneously cured in 20%–30% of patients, but the majority of infected patients fail to clear the virus and develop chronic persistent infection [2,3,4]. In addition to a combination regimen of pegylated interferon (IFN)-α and ribavirin, direct acting antiviral drugs (DAAs) against HCV have been developed, and a high rate of sustained virological response (SVR) has been achieved by using these antiviral drugs [5]. However, the high cost of these drugs results in limited access in developing nations where the disease burden is high; therefore, there is still a need for the development of a prophylactic vaccine.

Until now, the pathogenesis of HCV infection has not been clearly elucidated yet. Importantly, the detailed mechanism of innate immune activation by HCV and its implications for viral persistence and treatment response have not been clearly explained. Therefore, understanding HCV-host interactions and immune responses are important novel therapeutics with a higher barrier to viral resistance can be developed [6]. However, there is no established small animal model for the study of the entire life cycle of HCV infection and immunopathogenesis [7]. Severe combined immunodeficiency mice grafted with human hepatocytes are the only small animals that can be infected with HCV, although they cannot exert adaptive immune responses [8]. Recently, genetically-humanized mouse models are being developed to recapitulate the entire life cycle of HCV [9,10], but these models have restricted replication of HCV, limiting their utility.

As an experimental tool, development of cell culture-derived HCV (HCVcc) systems has dramatically facilitated HCV research over the last 10 years. Here, we review recent advances in the research on innate immune response in HCV infection and focus primarily on interferon response of host cells.

## 2. Cell Culture Systems of HCV Infection

It was not until 2005, more than 15 years after the discovery of HCV [11], that the first efficient cell culture model of HCV became available. The identification of a clinical isolate (genotype 2a) that replicates efficiently in Huh-7 hepatoma cells [12] made the first cell culture system possible. This isolate was obtained from a Japanese patient with fulminant HCV infection and was called JFH-1 [13,14,15]. Viral particles produced by the transfection of Huh-7 cells with *in vitro* transcribed JFH-1 RNA could infect naïve cells in cell culture and the liver of chimpanzees *in vivo* [14]. The HCV virion particles derived from the cell culture system were named “HCVcc” [13]. Until now, only JFH-1 spontaneously replicates in Huh-7 cells without adaptive mutations and releases infectious virus particles [14,15].

After the discovery of JFH-1-based HCVcc system, other HCV cell culture systems with various genotypes were established. For genotype 2 cell culture systems, J6cc (genotype 2a) [16] and J8cc/DH8cc/DH10cc (genotype 2b) [16,17] were developed. They replicated and propagated efficiently in Huh-7.5 cells, although they had adaptive mutations to facilitate their replication [16,17]. The first genotype 1a strain, H77-S, replicated and released infectious particles in Huh-7 cells and immortalized human hepatocytes, although the amount of released virus was lower than JFH-1 [18,19]. The Con1 (genotype 1b) cell culture system was also reported, but a very low level of replication has also limited its utility [20]. Recently, a new cell culture system of genotype 1a was developed. The TN genome with eight mutations (TNcc) [21] and H77C recombinant harboring 19 mutations (H77Ccc) replicated and spread efficiently in Huh-7.5 cells [22]. Recently, a cell culture system for infectious genotype 3a was also established by introducing adaptive mutations into the S310 strain [23].

HCVcc system has some limitations that should be considered. The most important limitation is the restricted availability of genotypes established in cell culture models. Currently, HCVcc systems for genotypes 4, 5, and 6 are unavailable. For genotypes 1 and 2, only specific patient clones have been propagated in cell culture systems. It should be noted, however, that a new host factor, SEC14L2 was recently reported to enable replication of non-adapted HCV in hepatoma cells [24]. New cell culture system utilizing SEC14L2-expressing hepatoma cells may overcome the limited availability of HCVcc system. Another limitation of the current HCVcc system is the non-polarized nature of Huh-7-based cells [25,26]. Hepatocytes are highly polarized in the liver and cell-to-cell transmission takes an important part in the spread of HCV, but the current HCVcc system does not reflect the viral spread occurring in the infected liver. In addition, Huh-7 cells are not fully differentiated [27] and, thus, have a defect in activation of the innate immune response by HCVcc infection [28].

In primary human hepatocytes (PHHs), replication and virus production by HCVcc infection have been reported [27], but it is difficult to obtain PHHs for experimental use. Immortalized human hepatocyte was reported to support HCV genome replication, virus assembly, and robust IFN response against the virus [19,29,30,31] and, thus, can be used as an alternative. Differentiated hepatocyte-like cells (DHCs) induced from pluripotent stem cells have also been used for HCVcc infection [32,33,34]. DHCs were found to mount an efficient innate immune response after HCVcc infection, including the production of chemokines and type III IFNs [33]. Recently, DHCs from adipose tissue-derived human mesenchymal stem cells (AT-hMSCs) were used for HCVcc infection [35], and the entry and replication of HCVcc were found to occur efficiently in DHCs from AT-hMSCs.

## 3. Interferon Response in HCV Infection

HCVcc infection systems provide a unique opportunity to study innate immune responses to HCV infection. Here, we focus mainly on recent advances in the study of interferon response in HCV infection.

### 3.1. Sensing of HCV by the Innate Immune System

In HCV-infected cells, viral RNA is sensed by retinoic acid-inducible gene I (RIG-I) and melanoma differentiation-associated protein 5 (MDA-5) in the cytoplasm and Toll-like receptor 3 (TLR3) in the endosome, which leads to downstream signaling that results in the induction of type III and I IFNs and other inflammatory cytokines [28,36,37,38,39]. Among these receptors, a role of MDA-5 in HCV sensing has remained controversial for several years, and it was recently proven that MDA-5 also participates in HCV sensing in the cytoplasm using HCVcc infection systems [28,36,40].

Intracellular signals from RIG-I, MDA-5, and TLR3 are transmitted via mitochondrial antiviral signaling protein (MAVS) and Toll/IL-1 receptor domain-containing adaptor inducing IFN-β (TRIF), respectively, which leads to the interferon regulatory factor-3 (IRF-3)-dependent induction of IFNs and NF-κB activation in HCV-infected cells [38,39]. Similar to other viruses, HCV uses several mechanisms to interfere with the induction of IFNs, particularly NS3/4A protease. NS3/4A cleaves MAVS, which leads to the impairment of signaling and IFN production in response to HCV RNA [41]. MAVS cleavage by NS3/4A has also been confirmed in HCV-infected liver tissue [42]. NS5A also contributes to immune evasion from the host. IFN-γ expression is inhibited in NS5A-transgenic mice after adenoviral challenge, meaning that NS5A plays an important role toward establishment of chronic HCV infection [43].

### 3.2. Endogenous Production of IFNs in HCV-Infected Cells

Despite HCV interference with the induction of IFNs, IFNs are endogenously produced by HCV-infected cells [28,29,44,45,46]. Both genotype 2a [28,29,44,45,46] and genotype 1a [29] were reported to activate intracellular interferon signaling pathways. IFNs are currently classified into three major classes: type I, type II, and type III. Among them, type I and III IFNs are considered innate immune response IFNs. Among type I IFNs, there are 13 IFN-αs, in addition to IFN-β, IFN-ω, IFN-ε, and IFN-κ [47]. IFN-λs (IFN-λ_1_ or IL-29; -λ_2_ or IL-28A; and -λ_3_ or IL-28B) are a new family of IFNs that have been designated as type III IFNs. Since the discovery of IFN-λs in 2003, their functions have been considered to overlap with type I IFNs because signaling via the IFN-λ receptor is similar to that via the IFN-α/β receptor. After binding to their receptors, type I and III IFNs initiate a signaling cascade through the Janus kinase (JAK)-signal transducer and activator of transcription (STAT) pathways. The cellular actions are then mediated by the induction of interferon-stimulated genes (ISGs) that have antiviral and/or immunomodulatory activity [48,49].

Recently, it was demonstrated that IFN-λs are major IFNs produced by HCV-infected cells [28,44,45,46]. IFN-λs activate the same JAK-STAT pathway as type I IFNs [48,49,50], thereby inducing a similar set of ISGs. Although the exact source of IFN-λs in HCV-infected liver remains to be clarified, it seems that the production of IFN-λs by HCV-infected hepatocytes results in the expression of ISGs, presumably through autocrine and/or paracrine signaling via the IFN-λ receptor [28,44,45,46].

Although HCV interferes with the induction of IFNs, continuous ISG up-regulation in HCV-infected liver has been demonstrated in chimpanzee models [51,52] and HCV-infected patients [53,54]. Interestingly, HCV RNA and ISG mRNA are detected simultaneously in hepatocytes from patients with chronic HCV infection [55]. This finding suggests that HCV infection potently stimulates the production of endogenous IFNs, which leads to ISG up-regulation in infected liver [55], and that HCV survives under the ISG up-regulation perhaps due to the protein kinase R (PKR)-mediated suppression of ISG protein translation [56,57].

As a rapid response to type III and I IFNs, IFN stimulated gene factor 3 (ISGF3), which consists of tyrosine-phosphorylated STAT1 (PY-STAT1), tyrosine-phosphorylated STAT2 (PY-STAT2), and IRF9, mediates the induction of numerous ISGs, including STAT1, STAT2, and IRF9 themselves [47]. Recently, it was demonstrated that prolonged induction of a set of ISGs is mediated by unphosphorylated ISGF3 (U-ISGF3), which is composed of unphosphorylated STAT1 (U-STAT1), unphosphorylated STAT2 (U-STAT2), and IRF9 [58,59]. The U-ISGF3 level is increased by sustained exposure to IFNs, and U-ISGF3 leads to enhanced expression of a set of ISGs (U-ISGF3-downstream ISGs, U-ISGs) [58]. In other words, there appear to be two phases of ISG expression following type III or I IFN stimulation. The initial rapid response is driven by the classical phosphorylated form of ISGF3, which is followed by a second, more prolonged response driven by U-ISGF3 [58].

In line with this report, we recently demonstrated that endogenous production of type III and I IFNs by HCV infection increases the levels of U-ISGF3, composed of U-STAT1, U-STAT2, and IRF9 proteins [60]. Using HCVcc infection systems with immune-competent liver cells such as PHHs and TLR3-transfected Huh7 cells, we demonstrated that U-ISGF3 induces the expression of U-ISGs [60].

### 3.3. Expression of ISGs and Responses to IFN Treatment

As mentioned above, ISGs induced by endogenous type III or I IFNs are up-regulated in HCV-infected liver [53,54,60,61,62]. Representative ISGs that are maintained at high levels of expression include ISG15, IFI27, IFI44, Mx1, and OAS-1 [53,54,60,61,62]. These ISGs are regulated not only by ISGF3 but also by U-ISGF3, and they are mainly antiviral [58,60]. We recently found that increased levels of U-STAT1, U-STAT2, and IRF9 are able to inhibit HCV RNA replication without exogenous IFN treatment [60]. This finding suggests that the sustained expression of ISGs by U-ISGF3 has antiviral activity against HCV in the infected liver but is insufficient to clear the virus.

Previously, it was demonstrated that patients with high levels of ISGs in their liver at baseline respond poorly to combined therapy with pegylated IFN-α and ribavirin [53,54,60,61,62,63,64]. Moreover, it has been shown that increased ISG expression at baseline is a stronger predictor of a poor response to pegylated IFN-α/ribavirin therapy than is the *IL28B* genotype [53]. Some reports have emphasized USP18 as a critical factor conferring unresponsiveness to exogenous IFN-α treatment by suppressing intracellular signaling [65,66,67,68]. However, the mechanism underlying the increase and maintenance of USP18 protein levels in HCV-infected liver has not been clearly elucidated. Recently, we found that prolonged exposure to IFN-λ up-regulates U-ISGF3 and U-ISGs, including ISG15, and that ISG15 causes the refractoriness to exogenous IFN-α treatment by stabilizing USP18 protein [60].

In 2013, the gene *IFNL4* was first described [69]. *IFNL4* expression is influenced by a germline dinucleotide frameshift variant located in exon 1 of *IFNL4* [69]. The *IFNL4*-∆G allele generates the full-length IFN-λ_4_ protein, whereas the *IFNL4*-TT allele does not create IFN-λ_4_ due to a premature stop [69]. The *IFNL4*-∆G allele is associated with a poor response to pegylated IFN-α/ribavirin therapy [69,70], and a recent study concluded that the *IFNL4*-∆G/TT genotype is the primary polymorphism underlying poor treatment response in HCV-infected patients [71]. Another study showed that *IFNL4*-∆G genotype is associated with high levels of ISGs and that hepatic levels of ISG15 in chronic hepatitis C are strongly associated with IFN-λ_4_ expression, suggesting that IFN-λ_4_ contributes to induction of ISGs in HCV-infected liver [72]. Forced expression of *IFNL4* gene up-regulates ISGs in PHHs and HepG2 cells [69,73], and has antiviral effects against HCV [74]. Recombinant IFN-λ_4_ protein activates the JAK-STAT pathway through binding to the IFN-λ receptor [75], evokes similar gene expression pattern to IFN-λ_3_ [76]. Future study using HCVcc infection systems will explain the mechanism of IFN-λ_4_ induction in HCV-infected cells and the effects of endogenous IFN-λ_4_ on both ISG induction and the response to exogenous IFN-α treatment.

### 3.4. Role of DUSP1 in HCV Infection

After HCV infection, the expression of some genes is down-regulated, and the expression of those genes tends to be further down-regulated in the liver of non-responders to IFN treatment [61,77]. One of the genes down-regulated in HCV-infected liver is dual specificity phosphatase 1 (DUSP1), a mitogen-activated protein kinase phosphatase (MKP) that de-phosphorylates mitogen-activated protein kinases (MAPKs) [78]. We demonstrated that silencing DUSP1 expression inhibits HCV replication in HCVcc-infected cells and HCV replicon cells by up-regulating antiviral ISGs [77]. DUSP1 silencing enhances the nuclear translocation of STAT1 and causes the induction of ISGs [77]. Although the detailed mechanism of DUSP1 down-regulation in HCV-infected liver remains to be elucidated, this serves as an example of how hosts regulate the expression of ISGs and restrict viral infection while bypassing endogenous IFN production.

### 3.5. Regulation of IFN-Induced MHC Class I Expression by HCV Infection

In virus-infected cells, viral peptides are processed and loaded onto major histocompatibility complex (MHC) class I molecules and presented to viral peptide-specific CD8^+^ cytotoxic T cells [79]. Recently, we demonstrated using an HCVcc system that IFN-induced up-regulation of MHC class I molecules is attenuated by HCV infection [57]. HCV RNA activates PKR, which phosphorylates the translation initiation factor eIF2α to block the translation of proteins, including ISGs [56] and MHC class I [57]. The attenuated expression of MHC class I by HCV infection causes a reduction of the effector functions of HCV-specific CD8^+^ T cells [57]. Before our study, several studies had investigated the effect of HCV proteins on MHC class I expression with conflicting results. The expression of MHC class I was not affected by overexpression of HCV proteins in one study [80], whereas another study showedup-regulation of MHC class I expression by the HCV core [81]. By using an HCVcc model, we were able to evaluate the effect of the whole life cycle of HCV infection on MHC class I expression, and we demonstrated the attenuation of IFN-induced MHC class I expression by HCVcc infection.

## 4. Conclusion

The isolation of the JFH-1 clone and the establishment of HCVcc infection systems made it possible to perform various studies on host-virus interactions and innate immune responses against HCV infection. Now is the era of DAAs, and HCVcc systems have greatly contributed to the advent of the DAA era. However, much remains to be resolved. Above all, the precise mechanism of interferon response and its paradoxical contribution to viral persistence should be elucidated. Novel cell culture models that closely mimic host responses with various clinical strains are the prerequisite for understanding the pathogenesis of HCV infection and clarifying the mechanism of viral persistence.
